# The causal relationship between risk of developing bronchial asthma and frailty: a bidirectional two-sample Mendelian randomization study

**DOI:** 10.3389/fmed.2023.1289026

**Published:** 2023-12-15

**Authors:** Xiao Ma, Haoran Xu, Jinghui Xie, Lu Zhang, Mengyao Shi, Zegeng Li

**Affiliations:** ^1^Anhui University of Chinese Medicine, Hefei, China; ^2^Department of Respiration, Wuhu Traditional Chinese Medicine Hospital, Wuhu, China; ^3^The First Affiliated Hospital of Anhui University of Chinese Medicine, Hefei, China

**Keywords:** asthma, frailty, Mendelian randomization analysis, causality, SNP, risk factor

## Abstract

**Background:**

A potential link between asthma and frailty has been suggested in previous studies. However, the nature of the causal relationship between these two conditions warrants further investigation. Therefore, this study assessed the bidirectional causality between asthma and frailty risk using two-sample Mendelian randomization (MR).

**Methods:**

The study data were obtained from the genome-wide association study (GWAS) dataset, with 337,159 samples representing asthma data and 175,226 samples representing frailty. The causal relationship between the two disorders was assessed by selecting the single nucleotide polymorphisms (SNPs), significantly associated with both asthma and frailty. The inverse variance weighting (IVW) method was used as the main analytical method to estimate the possible influence of causality. Sensitivity analysis was also performed using Mr-Egger intercept, funnel plot, “leave-one-out,” and Cochran Q test. In addition, potential mediators were investigated by risk factor analysis.

**Result:**

The IVW method showed an increased risk of frailty due to increased genetic susceptibility factors and the number of to asthma (OR = 2.325, 95%CI:1.958–2.761; *p* = 6.527498e-22), while no horizontal pleiotropy was observed for the Mr-Egger intercept (*p* = 0.609) and the funnel plot. The Cochran Q value was 72.858, *p* = 0.024, and there was heterogeneity in the Cochran Q-value. No single SNP was observed for “leave-one-out” that had a biasing effect on the instrumental variables. In addition, genetic susceptibility to frailty was associated with asthma (OR = 1.088, 95%CI:1.058–1.119; *p* = 4.815589e-09). In the causal relationship described above, several risk factors for frailty are complex, with asthma leading to a significant reduction in physical activity endurance.

**Conclusion:**

Our findings suggest a probable positive causal effect of asthma on the risk of developing frailty, potentially mediated by reduced physical activity endurance. At the same time, a causal relationship exists between frailty and asthma. Therefore, assessment strategies for frailty should include asthma and vice versa.

## 1 Introduction

The Global Asthma Initiative (GINA) defines asthma as a heterogeneous disease caused by chronic airway inflammation (1) due to complex gene–environment interactions. Chronic inflammation causes variable airway obstruction and increased responsiveness, resulting in recurrent symptoms of episodic chest tightness, wheezing, shortness of breath, and/or cough (2). Asthma is a prevalent chronic respiratory condition significantly impacting global health. Globally, asthma affects an estimated 300 million people and causes nearly 250,000 deaths each year (3). Projections indicated that by 2025, the number of individuals with asthma could increase by nearly 100 million (4), with older adults experiencing higher morbidity and mortality rates compared to the younger population (5), contributing substantially to the global disease burden. The presentation, diagnosis, and treatment of asthma in the older adults are further complicated by age-related changes in physiological function and immunology. As with asthma, frailty is recognized as a major public health problem. Frailty is a complex clinical condition associated with aging, prevalent across various demographics. As the physiological capacity of multiple organ systems declines due to population aging (6), the homeostatic reserves of individuals are reduced, leading to increased vulnerability to endogenous and/or exogenous stressors (7). In older adults, frailty increases the risk of progressive decline in several systems and adverse clinical outcomes (8). The increasing prevalence of asthma and frailty markedly impairs the quality of life and exacerbates the burden on healthcare systems due to the associated decline in patient productivity. Thus, both are attracting increasing attention.

The causal linkage between asthma and the condition of frailty has garnered considerable academic scrutiny yet remains a matter of debate. Previous studies conclude (9) that asthma is associated with an increased risk of frailty. In a cross-sectional observational study of older adults asthmatics with frailty (10), the investigators performed the Kihon Frailty Screening Inventory as well as the SF-36 scale, the Hyland scale, the Asthma Quality of Life Questionnaire (AQLQ), the Asthma Control Test (ACT), and the Asthma Control Questionnaire (ACQ). Among 69 outpatient participants with asthma aged over 65, 52 (75.4%) were classified as frail. Kihon Frailty Inventory was significantly associated with all scores obtained on the SF-36, Hyland Scale, AQLQ, ACT, and ACQ, and asthma and frailty were associated. In another study from China (11), 9,416 older adults asthma patients were included in a cross-sectional survey of the epidemiologic status and risk factors of frailty and pre-frailty. In this study, a high prevalence of frailty and pre-frailty was observed among older adults Chinese patients with asthma, suggesting that routine frailty assessment could benefit the management of asthma in this population. Evidence also supports the role of frailty as a significant risk factor for the development and progression of asthma (12). These findings underscore the potential for a bidirectional causal association between asthma and frailty, emphasizing the need to clarify this relationship to inform prevention and treatment strategies for both conditions. Nonetheless, observational studies are prone to confounders and reverse causality, making their results difficult to control (13), thus limiting our understanding of the relationship between the two diseases.

Mendelian randomization (MR) is an emerging statistical approach in epidemiological research that employs genetic variants [single nucleotide polymorphisms (SNPs)] as instrumental variables (IVs) to assess the observed causal relationships between exposure factors and clinical outcomes (14). Genetic variation follows the principle of random assignment of alleles to offspring and the natural causal effect of genetic variation on phenotype (15); hence, SNPs are not subject to potential confounders and are strongly correlated with exposure factors, making MR analyses less susceptible to confounders and reverse causation bias in observational studies (16). The two-sample MR estimates causal relationships, referring to data that measures exposure and outcomes in different datasets typically collected from genome-wide association studies (GWAS). Until now, two-sample MRs have been widely used to explore the causal relationship between different exposures and asthma (17, 18); however, no study has assessed the bidirectional causality between asthma and frailty.

Therefore, this study explored the potential causal relationship between asthma and frailty via a bidirectional two-sample MR study to provide a relevant theoretical basis for the relationship between asthma and frailty development.

## 2 Materials and methods

### 2.1 Study design

**Figure 1:**
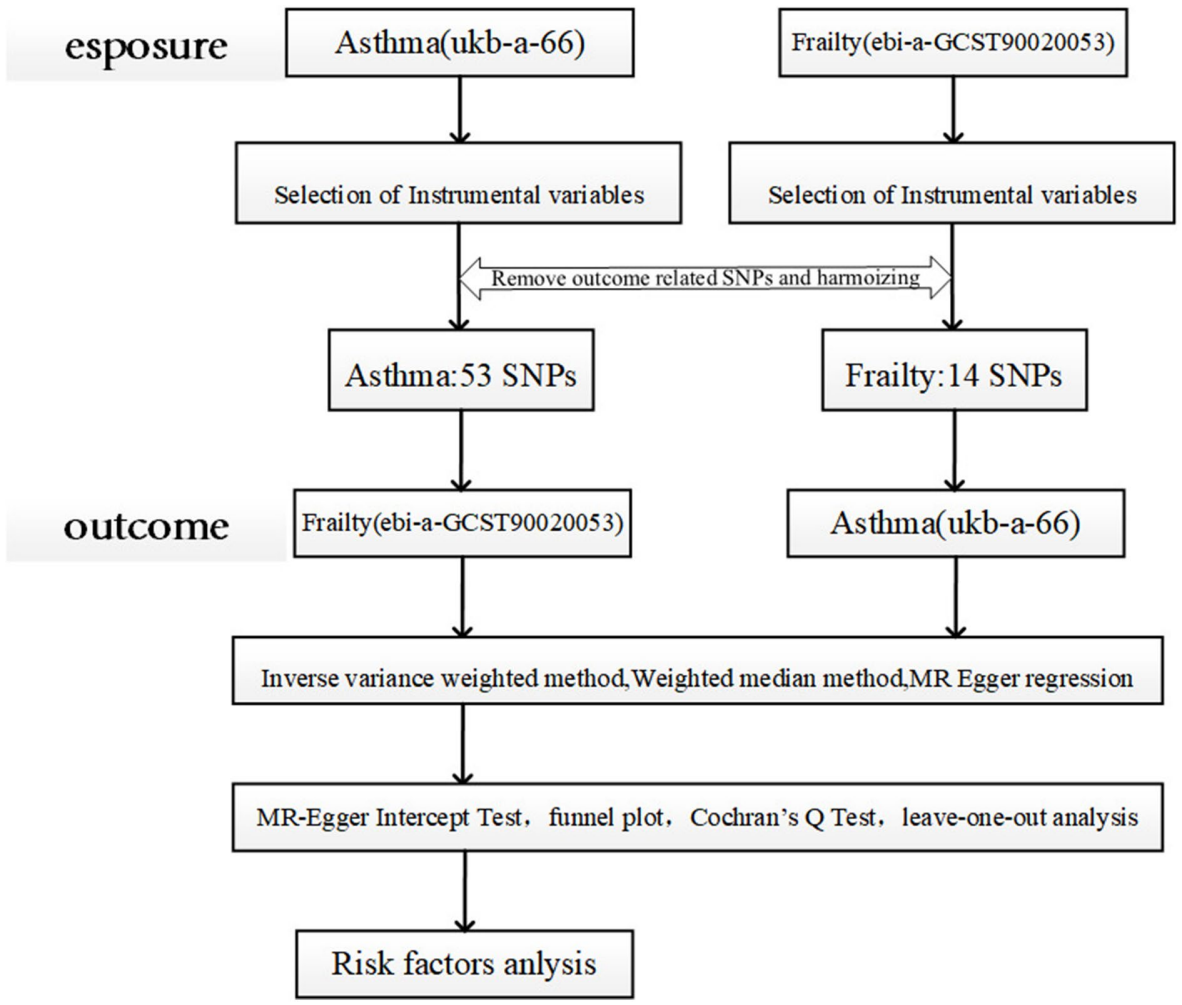
Schematic representation of the two-sample Mendelian randomization study design. MR analyses are required to meet the following criteria: (1) the instrumental variable must be strongly correlated with the exposure factor (assumption of correlation); (2) the instrumental variable must not be correlated with any confounders associated with the “exposure-outcome” association (assumption of independence); and (3) the instrumental variable affects the outcome variable only through the degree of exposure (assumption of exclusivity, also known as “no pleiotropy”). (3) the instrumental variable affects the outcome variable only through the degree of exposure (exclusivity assumption, also known as “no pleiotropy”) (22).ukb-a-66:UK Biobank-a-66.

In this study, a bidirectional two-sample MR analysis was performed to test the bidirectional causality between asthma and frailty using a publicly available GWAS pooled dataset, wherein no raw data were collected; therefore, no additional ethics committee approval or informed consent was required. Further sensitivity analyses were performed to test the reliability of the results. Furthermore, to investigate the potential genetic mechanisms underlying the combination of asthma and frailty, we considered several potential modifiers: body mass index (BMI), vitamin D,triglyceride, smoking, and physical activity (19–21). Here, all MR analyses were required to meet the three basic assumptions: association, independence, and exclusivity (22). Figure 1 presents a brief overview of the bidirectional MR analysis. This study complies with the STROBE-MR guidelines (23).

### 2.2 Data sources

The study data were mainly extracted from the Integrative Epidemiology Unit Open GWAS project website sample repository.^1^ The asthma dataset was derived from the UK Biobank, includes 337,159 individuals of European ancestry, and contains 39,049 cases and 298,110 controls. The frailty dataset was obtained from a dataset of 175,226 cases of European ancestry. The two-sample MR assumes independence between exposure data and outcome data. Therefore, data from asthma, having a significant overlap cohort with frailty, were excluded. The GWAS data corresponding to asthma were selected for analysis based on these criteria. Detailed information is shown in Table 1.

**Table 1:** Extraction of relevant data information from the Genome-Wide Association Studies database.

Diseases	Datasets	Sample size (people)	Number of SNPs	Sex	Population	Year
Asthma	ukb-a-66	337,159	10,894,596	Male and Female	European	2017
Fraitly	ebi-a-GCST90020053	175,226	7,589,717	Male and Female	European	2021

SNPs, single nucleotide polymorphisms.

### 2.3 Selection of instrumental variables

The first hypothesis of MR was that instrumental variables needed to be highly correlated with exposure factors; therefore, SNPs reaching the GWAS threshold (*p* < 5 × 10-8) were selected as preliminary results (24). The PLINK software was then employed to exclude SNPs in linkage disequilibrium (*r*^2^<0.001, genetic distance = 10,000 KB) (25). The selected instrumental variables were tested for weak instrumental variable bias by calculating the *F*-value; when the *F*-value was >10, the instrumental variables could be recognized as sufficient to overcome the weak instrumental tendency (26), further validating the hypothesis of the association between genetic variation and exposure factors, calculated as *F* = R^2^(N-2)/(1-R^2^) (27), where N is the sample size of the exposure factor, and *R*^2^ is the instrumental variable explaining the proportion of variance in the exposure factor.

Second, to satisfy the second MR hypothesis of no association of genetic variants with potential confounders, a query was performed using PhenoScanner (28),^2^ a comprehensive web-based database of genotype–phenotype associations, to determine whether there was no association between the screened SNPs and known confounders. We further investigated whether these SNPs were associated with potential risk factors such as BMI, vitamin D, triglycerides, smoking, and decreased exercise capacity. If SNPs associated with these potential confounders were found at the genome-wide significance level (*p* < 5 × 10^−8^), MR-presso analysis was performed to identify outliers. Finally, the remaining SNPs were extracted from the outcome data, ensuring that the effects of instrumental variables on exposure and outcome corresponded to the alleles with the same effects (29).

### 2.4 Mendelian randomization analysis

To avoid potential pleiotropic effects, three different MR methods (IVW, weighted median, and Mr-Egger) were used to assess the bidirectional causality between asthma and frailty. The primary analytical method to detect possible causality effects was IVW, and the results were supplemented by applying weighted median and Mr-Egger to assess the causal relationship between asthma and frailty in terms of OR values. Applying the MR-Egger method in this analysis yielded wider confidence intervals and non-significant *p*-values (30). However, the IVW method exhibited higher statistical efficacy than other MR methods and can provide robust results under a wider range of conditions (31). Should there be inconsistencies between the results of various MR analyses and prior research, it would necessitate a careful examination of the p-threshold for selecting instrumental variables, potentially leading to a reanalysis of the data (14).

Sensitivity analyses were performed to assess the reliability of the analyzed results, and potential heterogeneity and horizontal pleiotropy (influencing results through causal pathways rather than exposure) were observed. The Mr-Egger intercept test and funnel plots were used in this study to detect horizontal pleiotropy for all outcomes (*p* < 0.05 was considered to be horizontal pleiotropy) (32). Subsequently, the results’ stability was assessed using Cochran’s Q-test to test heterogeneity for statistically significant results. Heterogeneity was deemed present in the analysis at *p* < 0.05 (33). A “leave-one-out” was conducted to evaluate whether the observed causality was reliant upon or unduly influenced by a single SNP within the relationship (34).

### 2.5 Risk factors

The potential genetic mechanisms underlying the comorbidity of asthma and frailty were explored by analyzing several risk factors, including BMI, vitamin D, triglyceride levels, cigarette smoking, and decreased exercise capacity. Genetic information for BMI was obtained from the Genetic Investigation of Anthropomorphic Traits (GIANT) Consortium (35). The vitamin D dataset was retrieved from the UK Biospecimen Bank (UK Biobank) (36). For lipid metabolism, triglyceride data were collected from the UK Biobank (37). The dataset on smoking was extracted from the Gene Sequencing Consortium (GSCAN) (38). The physical activity datasets were obtained from the UK Biobank (UK Biobank) (39). Table 2 presents details of each dataset. Mendelian randomization was conducted with asthma as the exposure and the aforementioned risk factors as outcomes. IVW was used as the primary analysis method, and a statistically significant difference was *p* < 0.05.

**Table 2:** Data sources for frailty-related risk factors.

ID	Traits	Consortium	Sample size	Ancestry	Year	PubMed ID
ieu-a-835	BMI	GIANT	322,154	European	2015	25,673,413
ebi-a-GCST90000618	Vitamin D	UK Biobank	496,946	European	2020	32,242,144
ieu-b-111	Triglycerides	UK Biobank	441,016	European	2020	32,203,549
ieu-b-24	Smoking	GSCAN	341,427	European	2019	30,643,251
ebi-a-GCST006097	Physical activity	UK Biobank	377,234	European	2018	29,899,525

BMI, Body Mass Index; GIANT, genetic Investigation of Anthropom ometric Traits; GSCAN, GWAS Sequencing Consortium of Alcohol and Nicotine use.

### 2.6 Statistical analysis

The results of MR analysis are expressed as effect estimates (OR) and 95% confidence intervals (CI) that directly reflect the causal relationship between asthma and frailty, with a statistically significant *p* < 0.05. All analyses were executed with the two-sample MR package (version 0.5.7) and ggplot2 (version 3.4.2) in R (version 4.1.2).

## 3 Results

### 3.1 Identification of genetic instrumental variables

Instrumental variables exhibiting linkage disequilibrium, which could introduce bias, were excluded. Subsequently, 71 asthma-related SNPs meeting stringent selection criteria (genome-wide significance with *p* < 5 × 10^−8^ and low linkage disequilibrium with *r*^2^ < 0.001) were retrieved from the GWAS database. After data concordance and querying with PhenoScanner (see text footnote 2), confounders were excluded. Eighteen SNPs were detected as confounders and excluded. Finally, 53 SNPs were retained as valid instrumental variables for asthma, of which 12 were also associated with frailty (Table 3). The F statistics of all SNPs were > 10, indicating the absence of any weak bias in the study results and confirming the reliability of statistical test efficacy (Table 4).

**Table 3:** Extraction of Single Nucleotide Polymorphisms and *F*-values for instrumental variables of the asthma genes from the Genome-Wide Association Studies database.

Phenotype	SNP	EAF	*p*-value	Beta	SE	*N*	*R* ^2^	*F* value
Asthma	rs10836537	0.348423	4.24E-08	−0.00449479	0.000820122	175,226	1.71E-04	30.03702602
Asthma	rs10849819	0.32897	3.84E-08	−0.00455728	0.000828839	175,226	1.73E-04	30.23194661
Asthma	rs10912564	0.306329	4.73E-08	0.00464222	0.000850035	175,226	1.70E-04	29.82447884
Asthma	rs11071559	0.12784	3.66E-17	−0.00980791	0.00116432	175,226	4.05E-04	70.95831137
Asthma	rs11178649	0.407835	2.50E-10	−0.00502753	0.000794557	175,226	2.28E-04	40.03633074
Asthma	rs117710327	0.0670909	1.08E-20	−0.0148425	0.00159115	175,226	4.96E-04	87.0135549
Asthma	rs12123821	0.0480577	4.65E-29	0.0203649	0.00182	175,226	7.14E-04	125.2036041
Asthma	rs12365699	0.16674	8.60E-10	−0.0064442	0.00105062	175,226	2.15E-04	37.62198763
Asthma	rs12788104	0.687587	1.82E-08	0.00474152	0.000842392	175,226	1.81E-04	31.68120918
Asthma	rs12964116	0.0356748	6.12E-09	0.012218	0.00210159	175,226	1.93E-04	33.79864566
Asthma	rs13099273	0.507792	2.47E-16	−0.00649663	0.000792563	175,226	3.83E-04	67.18985778
Asthma	rs13208164	0.268003	2.53E-09	0.00526182	0.000882933	175,226	2.03E-04	35.51497551
Asthma	rs13355228	0.133025	9.08E-10	0.00704606	0.00115038	175,226	2.14E-04	37.51501045
Asthma	rs1684466	0.640335	1.75E-11	−0.00563833	0.000838297	175,226	2.58E-04	45.23766711
Asthma	rs16903574	0.0767099	1.52E-13	0.0110598	0.00149749	175,226	3.11E-04	54.54585179
Asthma	rs17227210	0.144129	1.28E-10	0.00715716	0.00111309	175,226	2.36E-04	41.34432758
Asthma	rs174535	0.350987	3.26E-11	−0.00542139	0.000817156	175,226	2.51E-04	44.01557665
Asthma	rs1837253	0.739738	8.09E-40	0.0117334	0.000888366	175,226	9.95E-04	174.4451656
Asthma	rs2197415	0.577297	4.24E-38	0.0101981	0.000790165	175,226	9.50E-04	166.5704648
Asthma	rs2296618	0.135639	2.90E-09	−0.00682889	0.00115018	175,226	2.01E-04	35.25036792
Asthma	rs2299012	0.1919	1.78E-25	0.0103275	0.000989948	175,226	6.21E-04	108.8330212
Asthma	rs2338819	0.289135	2.89E-09	−0.0051123	0.000860956	175,226	2.01E-04	35.25865906
Asthma	rs256866	0.116749	4.69E-13	0.00879709	0.00121603	175,226	2.99E-04	52.33407285
Asthma	rs28498223	0.279813	7.58E-13	0.00626785	0.000874311	175,226	2.93E-04	51.39257132
Asthma	rs2949669	0.407021	4.26E-13	−0.00575087	0.000793504	175,226	3.00E-04	52.524739
Asthma	rs3024664	0.939414	2.55E-13	0.0120316	0.00164448	175,226	3.05E-04	53.52844423
Asthma	rs3024971	0.10749	1.64E-23	−0.0125869	0.00125952	175,226	5.70E-04	99.86710731
Asthma	rs34290285	0.25536	2.89E-36	−0.0112363	0.000893421	175,226	9.02E-04	158.1719184
Asthma	rs35441874	0.247333	9.50E-27	−0.00971956	0.000907751	175,226	6.54E-04	114.6449109
Asthma	rs35570272	0.396247	7.45E-13	0.0057482	0.00080156	175,226	2.93E-04	51.42646951
Asthma	rs41283642	0.0337101	2.38E-10	−0.0136572	0.00215598	175,226	2.29E-04	40.12628248
Asthma	rs4247364	0.699883	1.02E-08	−0.00487025	0.000850413	175,226	1.87E-04	32.79727581
Asthma	rs4594881	0.342188	1.86E-08	−0.00462255	0.000821889	175,226	1.80E-04	31.6323853
Asthma	rs4739738	0.641396	3.91E-20	−0.00747427	0.000813201	175,226	4.82E-04	84.47667261
Asthma	rs479844	0.554816	2.52E-10	0.00495455	0.000783245	175,226	2.28E-04	40.01365394
Asthma	rs57347370	0.256642	1.28E-08	−0.00510659	0.000897556	175,226	1.85E-04	32.36934526
Asthma	rs58029167	0.27286	8.75E-14	0.00653444	0.000876073	175,226	3.17E-04	55.63283611
Asthma	rs59731494	0.431751	3.94E-09	0.00464042	0.000788289	175,226	1.98E-04	34.65282857
Asthma	rs61816766	0.0335796	2.61E-13	0.0163502	0.00223572	175,226	3.05E-04	53.48184218
Asthma	rs7072638	0.228411	6.77E-09	0.00537716	0.000927635	175,226	1.92E-04	33.60057496
Asthma	rs7134784	0.850068	2.57E-10	0.00691411	0.00109353	175,226	2.28E-04	39.97663255
Asthma	rs72823641	0.136984	4.71E-51	−0.0170251	0.00113259	175,226	1.29E-03	225.9586339
Asthma	rs75125788	0.0804599	1.51E-09	−0.00867192	0.00143485	175,226	2.08E-04	36.52687887
Asthma	rs7626218	0.395066	2.60E-11	−0.00532145	0.000798046	175,226	2.54E-04	44.46304121
Asthma	rs7734635	0.154436	4.59E-17	0.00908176	0.00108153	175,226	4.02E-04	70.51117363
Asthma	rs802731	0.270812	3.83E-10	0.00549251	0.000877267	175,226	2.24E-04	39.1988359
Asthma	rs891058	0.295608	2.70E-13	−0.00624809	0.000854858	175,226	3.05E-04	53.41972349
Asthma	rs912131	0.704195	6.14E-17	0.00712811	0.000852343	175,226	3.99E-04	69.93829354
Asthma	rs919826	0.490716	1.94E-08	−0.00439323	0.000782049	175,226	1.80E-04	31.556948
Asthma	rs9344188	0.550587	1.46E-08	0.00444739	0.000784876	175,226	1.83E-04	32.10726959
Asthma	rs947591	0.477263	1.67E-08	−0.00447686	0.000793276	175,226	1.82E-04	31.84882682
Asthma	rs981625	0.0650121	2.29E-09	0.00943578	0.00157898	175,226	2.04E-04	35.71062062
Asthma	rs992969	0.747765	1.54E-56	−0.0142432	0.000898787	175,226	1.43E-03	251.1288592

SNPs, single nucleotide polymorphisms; SE, standard error; EAF, effect allele frequency.

**Table 4:** Extraction of Single Nucleotide Polymorphisms and *F*-values for instrumental variables of the frailty genes from the Genome-Wide Association Studies database.

Phenotype	SNP	EAF	*p*-value	Beta	SE	*N*	*R* ^2^	*F* value
Frailty	rs12739243	0.2206	1.28E-09	−0.0242	0.004	175,226	2.09E-04	36.60208223
Frailty	rs1363103	0.38	2.23E-08	−0.0191	0.0034	175,226	1.80E-04	31.55759828
Frailty	rs17612102	0.5933	2.85E-08	0.0187	0.0034	175,226	1.73E-04	30.24965473
Frailty	rs2071207	0.478	1.47E-08	−0.0187	0.0033	175,226	1.83E-04	32.1107446
Frailty	rs2396766	0.4725	1.22E-09	0.0201	0.0033	175,226	2.12E-04	37.09875011
Frailty	rs3959554	0.4177	1.74E-08	0.0189	0.0034	175,226	1.76E-04	30.90016634
Frailty	rs4146140	0.3811	6.83E-09	−0.0198	0.0034	175,226	1.94E-04	33.91310773
Frailty	rs4952693	0.3734	1.47E-08	−0.0194	0.0034	175,226	1.86E-04	32.55672182
Frailty	rs56299474	0.1733	3.94E-08	0.0241	0.0044	175,226	1.71E-04	30.00017411
Frailty	rs583514	0.5111	1.65E-09	0.0199	0.0033	175,226	2.07E-04	36.36413958
Frailty	rs8089807	0.1866	6.50E-09	−0.0248	0.0043	175,226	1.90E-04	33.26300595
Frailty	rs82334	0.3177	3.13E-10	−0.0223	0.0035	175,226	2.32E-04	40.5946387

SNPs, single nucleotide polymorphisms; SE, standard error; EAF, effect allele frequency.

### 3.2 Mendelian randomization analysis

**Figure 2:**
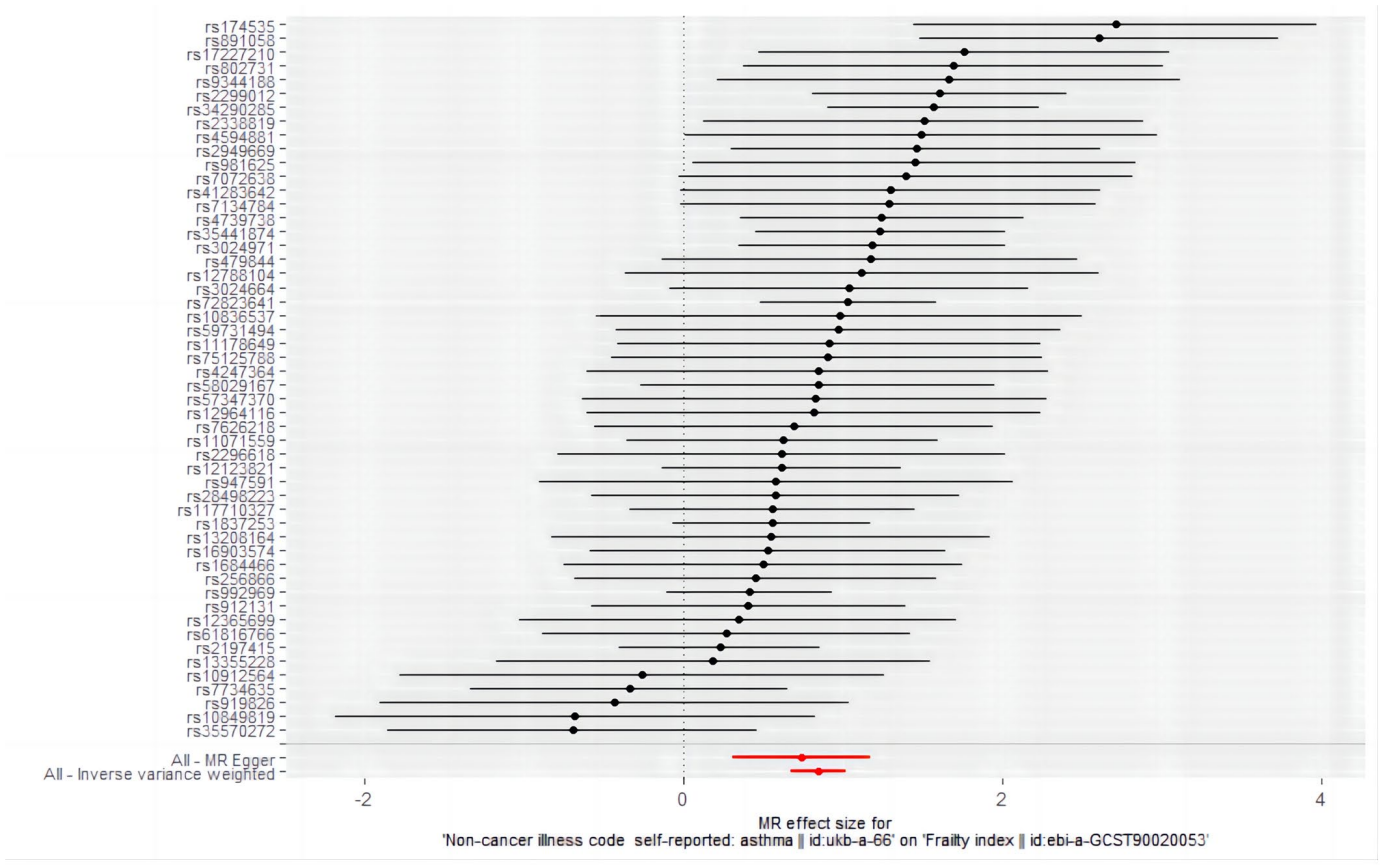
Forest plot of the causal effects of single nucleotide polymorphisms associated with asthma on frailty. Mendelian randomization estimates the effect size and data are expressed as effect values with 95% CI. The significance of red lines are MR results of MR-Egger test and IVW method.

**Figure 3:**
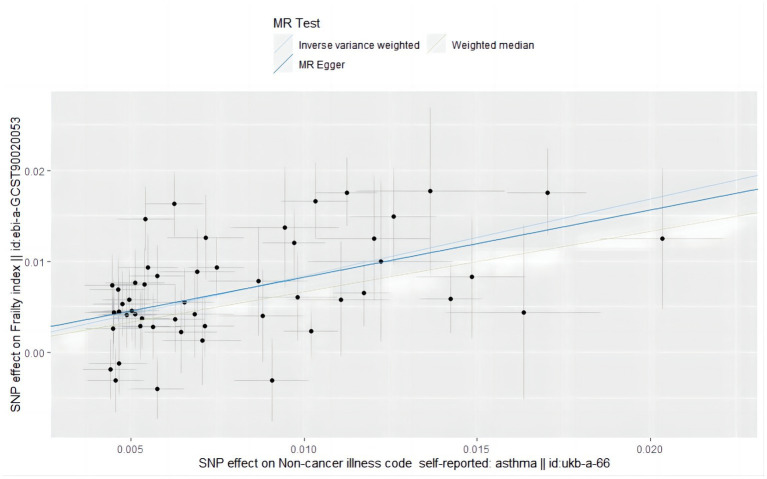
Scatter plot of the causal effect of single nucleotide polymorphisms associated with asthma on frailty in a Mendelian randomization study.

The analysis examined the genetic susceptibility to asthma as a potential causal factor in frailty development, revealing a strong and positive association (Figure 2). The IVW method revealed that genetic predisposition to asthma increased the risk of developing frailty (OR = 2.325, 95% CI: 1.958–2.761; *p* = 6.527498e-22) (Table 5), and the same estimate was observed in the weighted median analysis (OR = 1.943, 95% CI: 1.533–2.462; *p* = 3.921789e-08). In the Mr-Egger analysis (OR = 2.095, 95% CI: 1.358–3.232; *p* = 1.580040e-03), a consistent direction of effect was observed (see Figure 3 for details), in line with the results of previous studies (40).

**Table 5:** Results of MR analysis for the effect of asthma on frailty.

MR method	N.SNPs	Beta	SE	OR (95%CI)	*P*-value	Heterogeneity test		Pleiotropy test
						Cochran’s Q	*P*	*P* intercept
Inverse variance weighted	53	0.844	0.088	2.325 (1.958–2.761)	6.527498e-22	72.858	0.024	0.609
Weighted median	53	0.664	0.121	1.943 (1.533–2.462)	3.921789e-08			
MR Egger	53	0.739	0.221	2.095 (1.358–3.232)	1.580040e-03	72.474	0.021	

MR, Mendelian randomization; SNPs, single nucleotide polymorphisms; IVW, inverse variance weighted; OR, odds ratio; CI, confidence interval; SE standard error; N.SNPs: number of SNPs used in MR.

**Figure 4:**
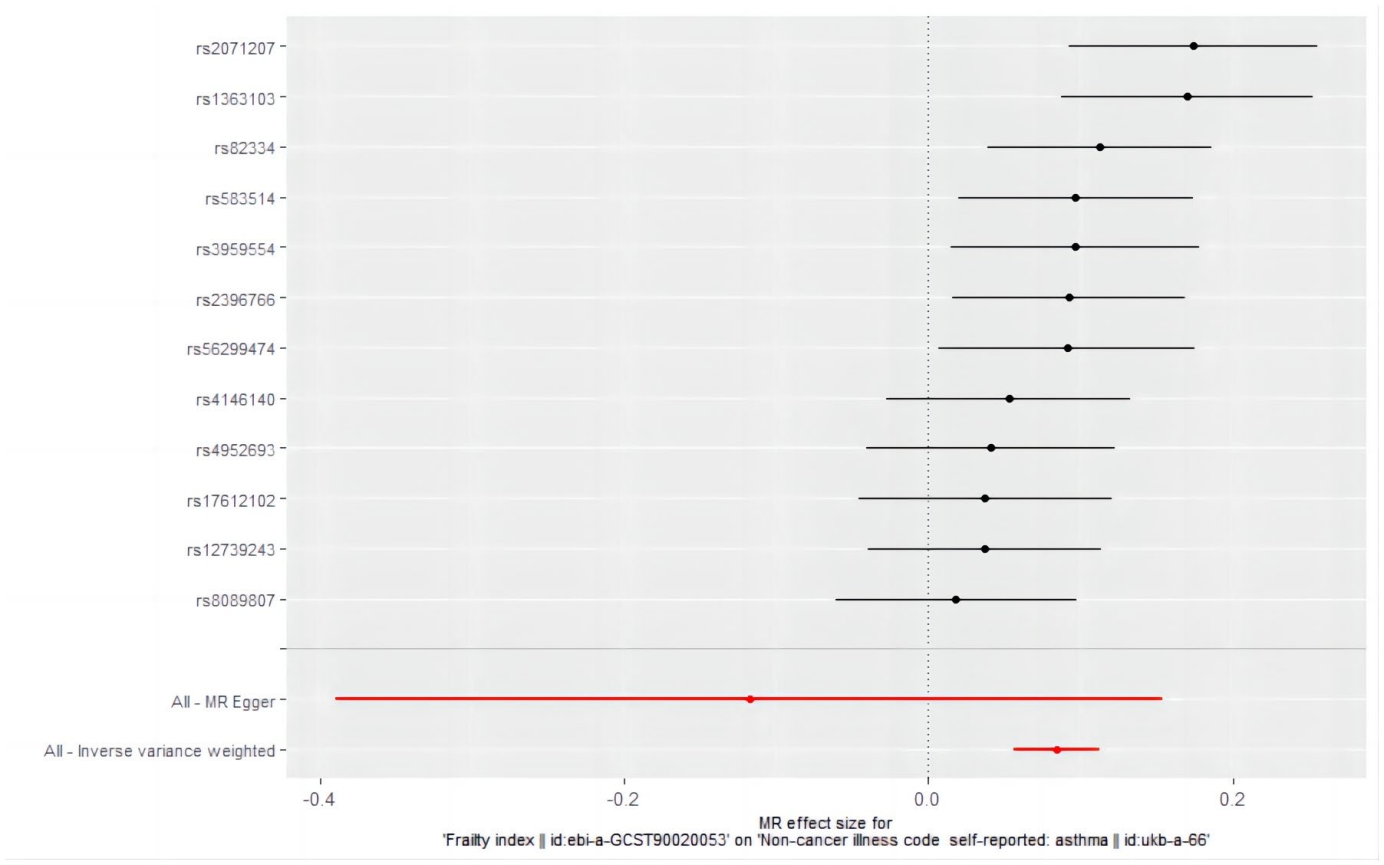
Forest plot of the causal effects of single nucleotide polymorphisms associated with frailty on asthma. Mendelian randomization estimates the effect size and data are expressed as effect values with 95% CI. The significance of red lines are MR results of MR-Egger test and IVW method.

**Figure 5:**
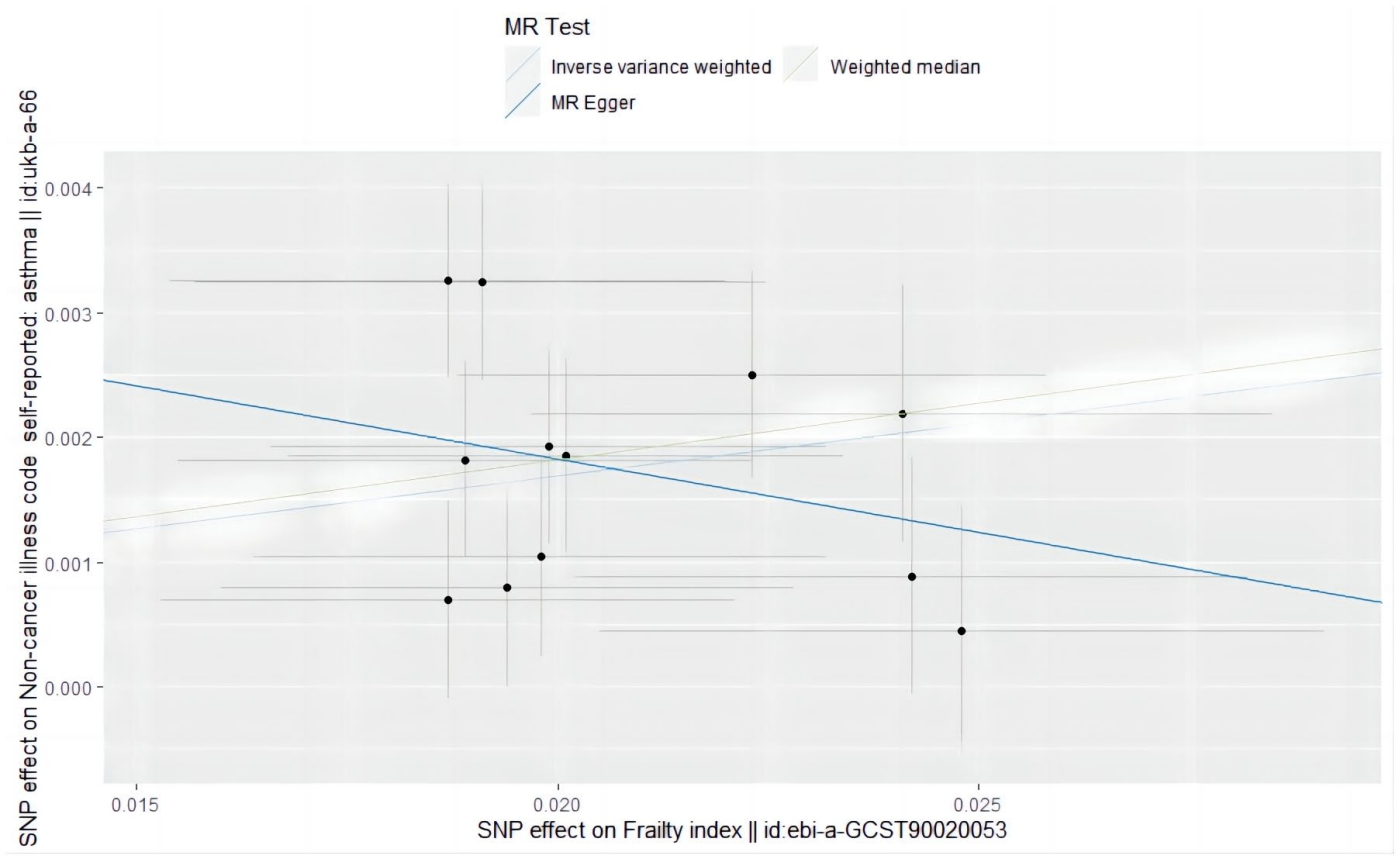
Scatter plot of the causal effect of single nucleotide polymorphisms associated with frailty on asthma in a Mendelian randomization study.

According to the IVW analysis under the random-effects model with frailty as the exposure factor and asthma as the outcome factor, a causal relationship was observed between frailty and asthma (OR = 1.088, 95% CI: 1.058–1.119; *p* = 4.815589e-09) (Figure 4). Weighted median analysis revealed the same estimate (OR = 1.095, 95% CI: 1.057–1.135; *p* = 4.723029e-07) (Table 6). The results of the Mr-Egger analysis (OR = 0.889, 95% CI: 0.677–1.166; *p* = 4.154820e-01) were not statistically significant, probably due to the large Mr-Egger’s confidence interval and lower effectiveness (Figure 5), suggesting a causal relationship between genetic susceptibility to frailty and asthma.

**Table 6:** Results of MR analysis for the effect of frailty on asthma.

MR method	N.SNPs	Beta	SE	OR (95%CI)	*P*-value	Heterogeneity test		Pleiotropy test
						Cochran’s Q	*P*	*P* Intercept
Inverse variance weighted	12	0.085	0.014	1.088 (1.058–1.119)	4.815589e-09	16.681	0.118	0.173
Weighted median	12	0.091	0.018	1.095 (1.057–1.135)	4.723029e-07			
MR egger	12	−0.118	0.139	0.889 (0.677–1.166)	4.154820e-01	13.728	0.186	

MR, Mendelian randomization; SNPs, single nucleotide polymorphisms; IVW, inverse variance weighted; OR, odds ratio; CI, confidence interval; SE standard error; N.SNPs, number of SNPs used in MR.

### 3.3 Sensitivity analysis

**Figure 6:**
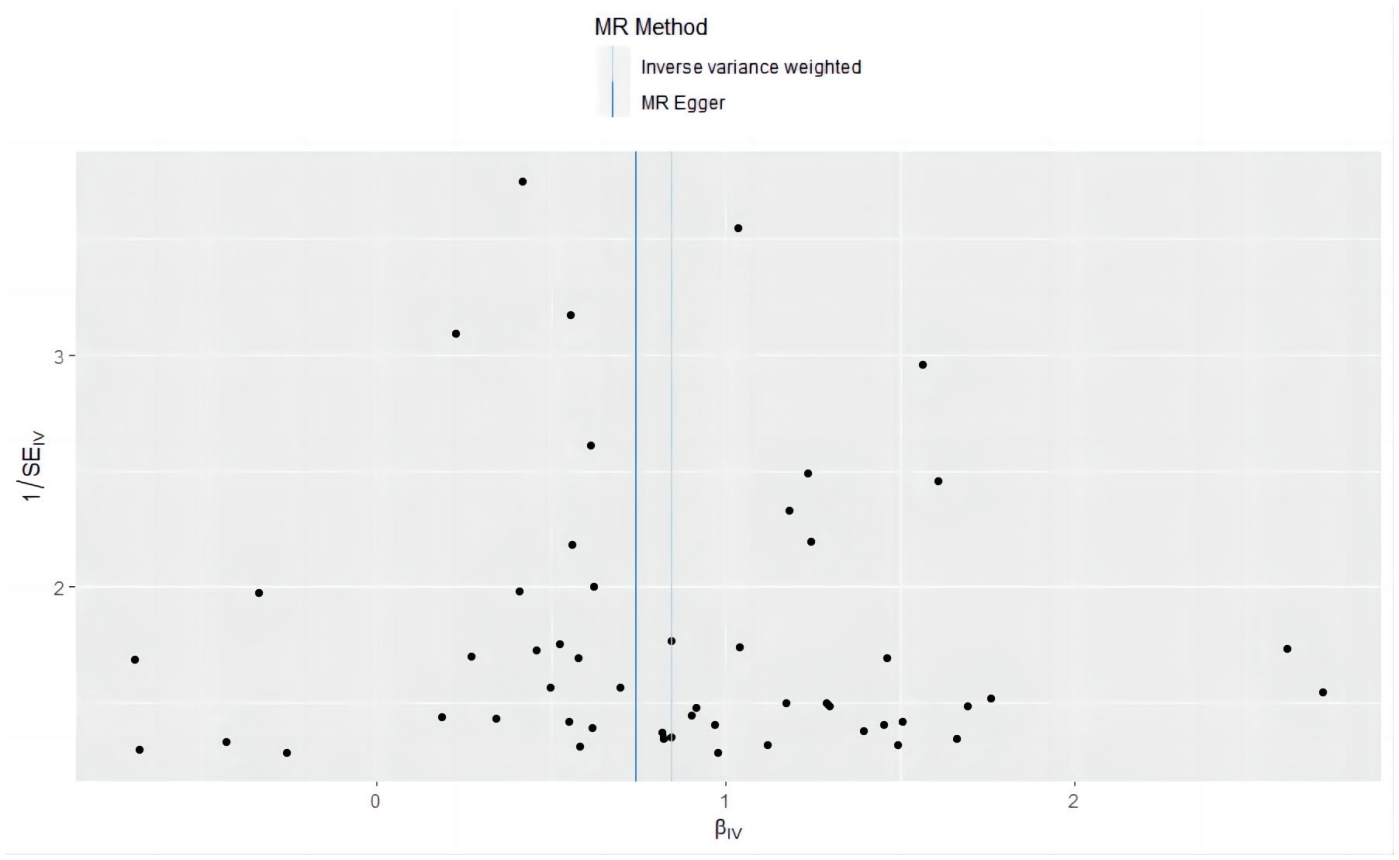
Funnel plot of causal effects in a Mendelian randomization study of asthma as an exposure factor versus frailty as an outcome factor.

**Figure 7:**
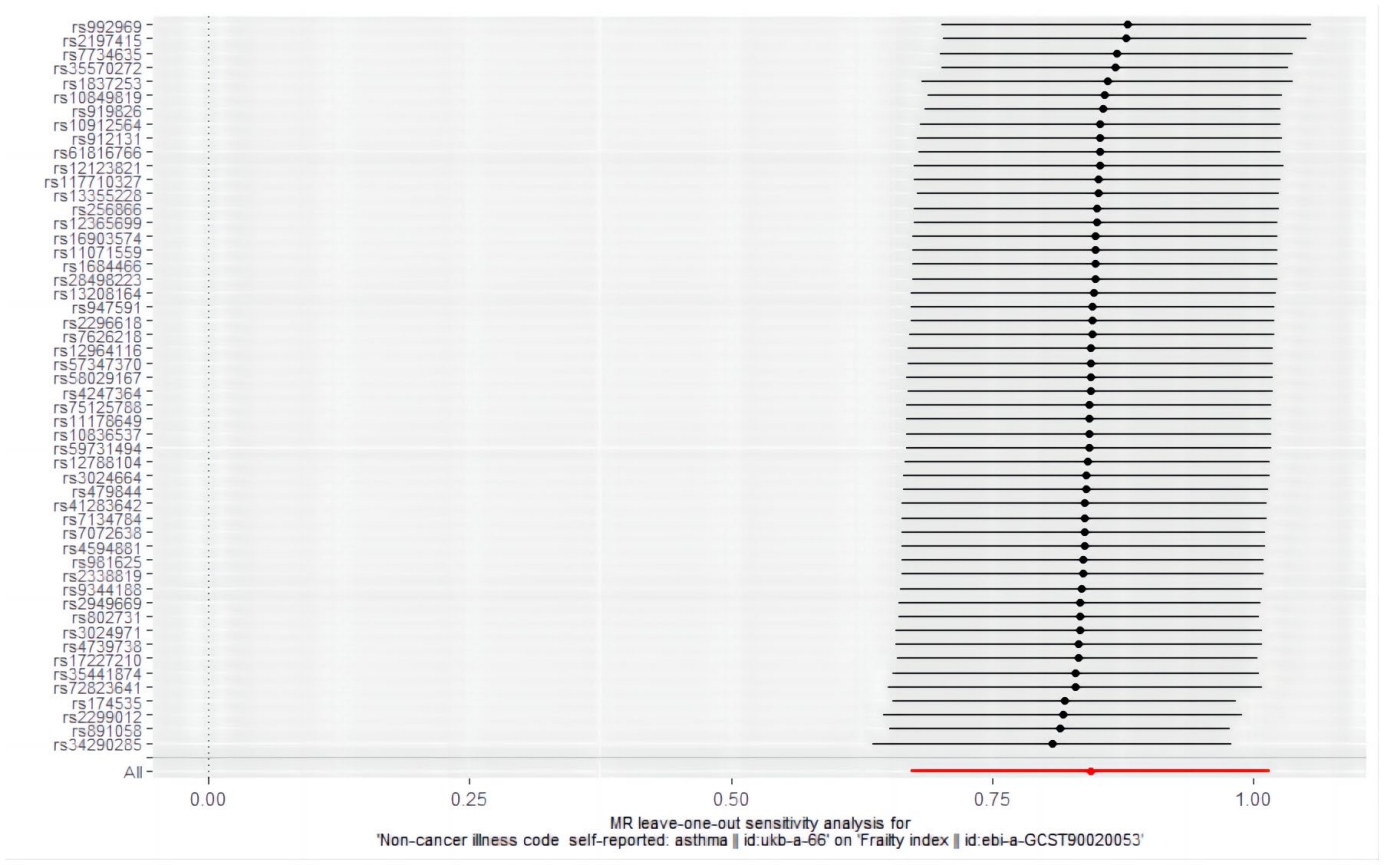
Results of sensitivity analysis of “leave-one-out” in a Mendelian randomized study of asthma-induced frailty.

A series of sensitivity tests, including Mr-Egger intercept, funnel plot, Mr-PRESSO detection, Cochran Q test, and leave-on-out analysis, were conducted to evaluate the accuracy of the positive results. In the MR analysis with asthma as the exposure factor, the *p*-value of the Mr-Egger intercept was 0.609. The MR-PRESSO test (*p* = 0.734) and the symmetric funnel plot (Figure 6) indicated no evidence of horizontal pleiotropy, which might otherwise suggest bias in the asthma-related findings. For further analysis, we used Cochran’s Q test, and the included SNPs showed heterogeneity in the results (*Q* value 72.858, *p* = 0.024) (Table 5). Despite the observed heterogeneity (*Q* value 72.858, *p* = 0.024, Table 5), the stability of the MR findings remains robust, supported by applying an IVW analysis within a random-effects model that accounts for this variability. In addition, the “leave one out” confirmed the robustness of our results, demonstrating that no individual SNP disproportionately influenced the causal estimates, proving that the effect used to evaluate causality is not dependent on any single genetic instrument variable to drive the results, indicating that the results were reliable (Figure 7).

In the MR analysis with frailty as the exposure factor, the *p* value of the Mr-Egger intercept was 0.173. The included SNPs were detected by Mr-PRESSO (*p* = 0.223), so there was no level of pleiotropy. Cochran’s Q test showed no heterogeneity between the two diseases (*Q* value 16.681, *p* = 0.118) (Table 6).

### 3.4 Risk factor analysis

**Figure 8:**
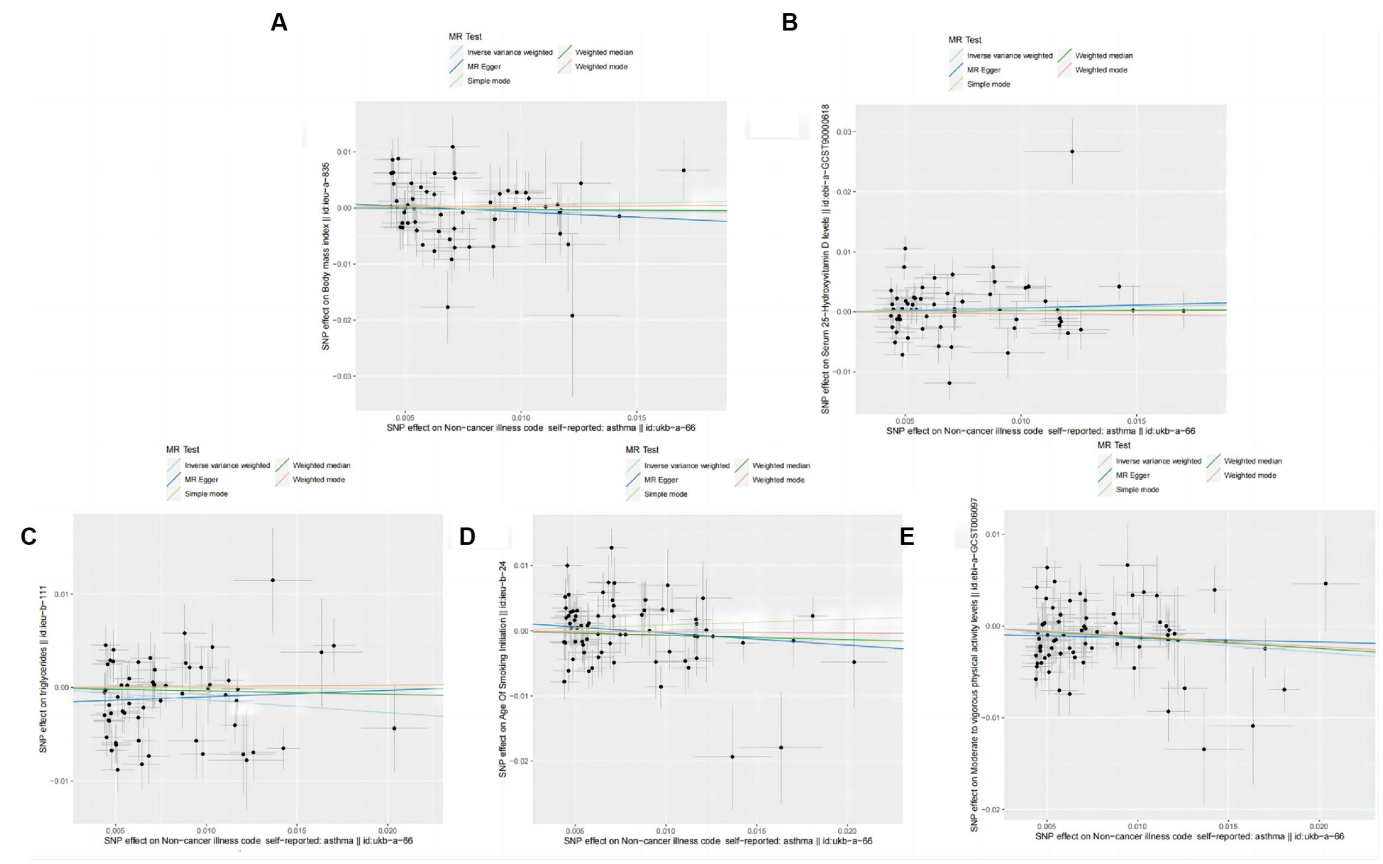
Scatter plot of the causal effect of single nucleotide polymorphisms associated with asthma on risk factors for frailty in the Mendelian randomization study. **(A)** Forest plot of the causal effect of single nucleotide polymorphisms associated with asthma on BMI. **(B)** Forest plot of the causal effect of single nucleotide polymorphisms associated with asthma on Vitamin D. **(C)** Forest plot of the causal effect of single nucleotide polymorphisms associated with asthma on Triglycerides. **(D)** Forest plot of the causal effect of single nucleotide polymorphisms associated with asthma on Smoking. **(E)** Forest plot of the causal effect of single nucleotide polymorphisms associated with asthma on physical activity.

**Figure 9:**
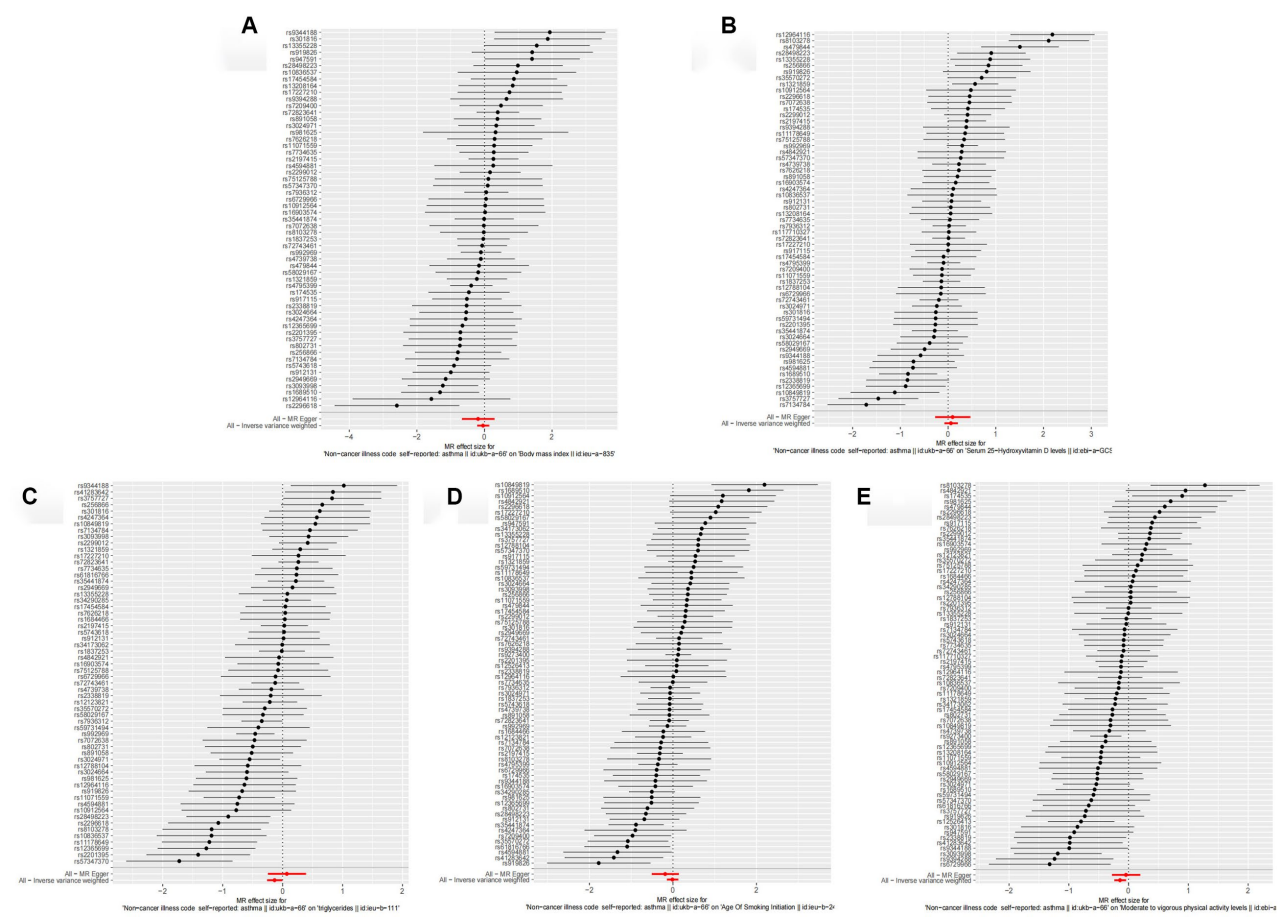
Forest plot of the causal effect of single nucleotide polymorphisms associated with asthma on risk factors for frailty. Mendelian randomization estimated the effect size and the data are expressed as effect values with 95% CI. The significance of the red line is the Mr-Egger test and the MR results of the IVW method. **(A)** Scatter plot of the causal effect of single nucleotide polymorphisms associated with asthma on BMI. **(B)** Scatter plot of the causal effect of single nucleotide polymorphisms associated with asthma on Vitamin D. **(C)** Scatter plot of the causal effect of single nucleotide polymorphisms associated with asthma on Triglycerides. **(D)** Scatter plot of the causal effect of single nucleotide polymorphisms associated with asthma on Smoking. **(E)** Scatter plot of the causal effect of single nucleotide polymorphisms associated with asthma on Smoking physical activity.

MR analysis was used to further investigate the causal relationship between asthma and the common risk factors, including BMI, Vitamin D, Triglycerides, Smoking, and physical activity for frailty. Table 7 and Figure 8 show a negative causal association between the genetic susceptibility to asthma with physical activity (OR = 0.866, 95% CI: 0.788–0.953; *p* = 0.003) and asthma resulted in a significant reduction in physical activity endurance (Figure 9). Overall, the risk factor analysis shows that decreased physical activity endurance may be responsible for the increased risk of frailty due to asthma. However, the results of the analysis concluded that genetic susceptibility to asthma was not associated with BMI (OR = 0.960, 95% CI: 0.809–1.138; *p* = 0.635), Vitamin D (OR = 1.061, 95% CI: 0.927–1.216; *p* = 0.390), Triglycerides (OR = 0.808, 95% CI: 0.635–1.030; *p* = 0.085), Smoking (OR = 0.988, 95% CI: 0.867–1.125; *p* = 0.856).

**Table 7:** Results of the analysis of common risk factors for asthma and frailty.

Exposure	Outcome	IVW		Cochran Q test		Mr-Egger intercept tests	
		OR (95%CI)	*P*	*Q* value	*P*	Mr-Egger intercept	*P*
Asthma	BMI	0.960 (0.809–1.138)	0.635	71.853	0.075	0.001	0.516
Asthma	Vitamin D	1.061 (0.927–1.216)	0.390	172.297	8.637478e-13	−0.0003	0.836
Asthma	Triglycerides	0.808 (0.635–1.030)	0.085	833.985	1.023915e-131	−0.001	0.577
Asthma	Smoking	0.988 (0.867–1.125)	0.856	125.400	3.911886e-05	0.002	0.245
Asthma	Physical activity	0.866 (0.788–0.953)	0.003	110.633	0.002	−0.001	0.363

BMI, Body mass index; IVW, inverse variance weighted.

## 4 Discussion

The rapid rise in the prevalence of asthma and frailty has led to an increasing overlap between patients with asthma and those with frailty (41). The quality of life of many such patients is severely affected by these comorbid conditions, necessitating a clearer understanding of their causal relationship to improve prevention and treatment strategies. The results of observational studies in clinical research can easily be confounded by factors that are difficult to control, resulting in a lack of established causality. Randomization in randomized controlled trials (RCTs) avoids confounding and minimizes selection bias, representing the highest level of evidence, yet RCTs are also constrained by practical limitations. First, RCTs are financially costly and time-consuming, particularly for rare outcomes or conditions requiring long-term follow-up. Second, RCTs may not always yield therapies that selectively affect the intended risk factors, and crucially, random assignment of many risk factors is often not feasible due to practical or ethical constraints (42). MR offers a time-and cost-effective alternative for uncovering causal relationships (43). This bidirectional two-sample MR analysis utilized datasets from the GWAS database and employed multiple methods to examine the possible causal relationship between asthma and frailty systematically. Our observations indicate a significant association between genetic susceptibility to asthma and frailty, with reverse MR analysis supporting a causal link from frailty to genetic predisposition for asthma. To our knowledge, this is the first study to apply an MR framework to ascertain a causal link between asthma and frailty.

Asthma ranks as the second most prevalent chronic respiratory disease in the older adults, following COPD, and is also the second leading cause of death from chronic respiratory conditions (44). Scant observational clinical studies addressed asthma and frailty, yet our findings concur with these earlier studies (10). In an observational clinical study from Japan (45), 37% of the 203 older adults asthmatic patients (60 years or older) attending an outpatient clinic had comorbid conditions. Another cross-sectional study analyzing 34,403 eligible participants (aged ≥40 years) from the 1999–2018 National Health and Nutrition Examination Survey cycles found a 32.3% prevalence of concurrent asthma and frailty (46). This study identified an increased risk of frailty among patients with asthma, which may be partly ascribed to the inherent disease characteristics of asthma. Since asthma involves chronic airway inflammation, the airway epithelium acts as both a mediator and target, leading to airway remodeling and obstruction (47). Furthermore, the chronic inflammation observed in asthmatic patients is present in the respiratory tract and also manifests as systemic inflammation. Inflammation causes the increase in the levels of peripheral blood eosinophils, total blood IgE, and type 2 cytokines by regulating growth factors and increasing catabolism (48). Frailty is a state of diminished physiological reserves with a manifestation of aging in an aging population. Aging alters the immune response, leading to a chronic proinflammatory state and increased susceptibility to airway infection (49). As epigenetics change, airway epithelial dysfunction and inflammatory cytokine activity are more pronounced in immunosenescent, older patients with asthma, who are at higher risk for poor clinical outcomes (50). Thus, chronic systemic inflammation may be an important cause of frailty in older adults with asthma. This study also explored potential risk factors associated with the combined presence of asthma and frailty, such as BMI, vitamin D levels, triglycerides, smoking habits, and physical activity. It was found that decreased physical activity endurance may play a moderating role (Table 6), consistent with several observational studies (51), which reported that physical activity levels are negatively correlated with asthma severity. Since decreased physical activity endurance is a recognized cause of frailty, physical activity may play a key intermediate role in the asthma-frailty pathway.

To summarize, frailty, as a multi-comorbid and chronic disease, places a heavy burden on patients and their families and poses challenges to health systems worldwide. For patients with established asthma, attention should be paid to the changes in their ability to perform physical activity, adopt a more rigorous assessment method, formulate appropriate public health prevention strategies, and reduce asthma patients’ frailty burden to prevent asthma patients from becoming weaker and disabled.

Reverse MR analysis confirmed a possible causal relationship between frailty and asthma, suggesting that these conditions may share similar pathophysiological mechanisms. Definitions of frailty are often associated with aging and chronic disease, strongly influencing health and function in later life (52). Frail older adults may experience swallowing dysfunction, increasing the risk of aspiration and choking, potentially leading to respiratory disease. However, the complex interplay regulating asthma in frail patients is not fully understood, and only some research has been published in this area over the past few years. A cross-sectional clinical study (12) is underway in patients over 60 to assess the multidimensional relationship between frailty phenotypes and asthma. In another cross-sectional study from South Korea (53), the researchers examined frailty-related diseases among the older adults in the community. They found a close relationship between frailty and hypertension, diabetes, arthritis, asthma, and other diseases. It is necessary to study the mechanism of frailty and asthma further to identify, manage, and prevent the occurrence and development of asthma and achieve healthy aging.

However, this study also had some limitations. First, it was based on European populations, and whether there are genetic differences among other ethnic groups, countries, and regions cannot be determined. Second, this study only analyzed the relationship between asthma and frailty. The absence of comprehensive clinical information precluded further subgroup analyses on pre-frailty and frailty, thereby limiting the ability to delineate their precise causal relationships. Third is the limitation of GWAS data; more SNPs are needed as variable tools to repeat MR research, improve test efficiency, and present more data to explore potential mechanisms and provide practical means of preventing and treating frailty.

## 5 Conclusion

This investigation, employing a bidirectional two-sample MR approach, has established a causal link between asthma and frailty. Therefore, it is imperative to enhance the assessment and management of frailty among patients with asthma to mitigate the incidence of adverse outcomes. Furthermore, this research contributes novel insights into the investigation and prevention of frailty in the context of asthma and proposes viable evaluation methodologies for forthcoming studies, thereby facilitating the early identification, diagnosis, and management of these conditions.

## Data availability statement

Publicly available datasets were analyzed in this study. This data can be found here: this study is based on the IEU Open GWAS project website summary data sample repository (https://gwas.mrcieu.ac.uk/), which are derived from publicly available studies. Ethical approval was obtained for all origin studies. The frailty dataset was obtained from https://gwas.mrcieu.ac.uk/datasets/ebi-a-GCST90020053. The Asthma dataset was obtained from https://gwas.mrcieu.ac.uk/datasets/ukb-a-66/.

## Ethics statement

This study was analyzed using the publicly available GWAS pooled dataset, and in accordance with legal and institutional requirements, human participant studies do not require ethical review and approval nor written informed consent.

## Author contributions

XM: Conceptualization, Data curation, Formal analysis, Methodology, Project administration, Software, Validation, Writing – original draft, Writing – review & editing. HX: Investigation, Software, Writing – review & editing. JX: Data curation, Investigation, Methodology, Writing – review & editing. LZ: Investigation, Methodology, Validation, Writing – original draft, Writing – review & editing. MS: Conceptualization, Data curation, Writing – original draft, Writing – review & editing. ZL: Conceptualization, Data curation, Formal analysis, Funding acquisition, Investigation, Resources, Writing – original draft, Writing – review & editing.

## Footnotes

^1^ https://gwas.mrcieu.ac.uk/

^2^ http://www.PhenoScanner.medschl.cam.ac.uk
